# Association between pre-pandemic wealth and material hardships during the COVID-19 pandemic: how racial and ethnic wealth inequities shape household vulnerability to national crises

**DOI:** 10.1093/haschl/qxaf078

**Published:** 2025-04-09

**Authors:** Alexandra Skinner, Nicole C McCann, Chanelle J Howe, Kathryn M Leifheit, Lorraine T Dean, Yareliz Diaz, Catherine K Ettman, Julia Raifman, Paul R Shafer

**Affiliations:** Department of Epidemiology, Brown University School of Public Health, Providence, RI 02903, United States; Department of Health Law, Policy and Management, Boston University School of Public Health, Boston, MA 02118, United States; Department of Epidemiology, Brown University School of Public Health, Providence, RI 02903, United States; Department of Epidemiology, Boston University School of Public Health, Boston, MA 02118, United States; Department of Pediatrics, University of California Los Angeles David Geffen School of Medicine, Los Angeles, CA 90095, United States; Department of Epidemiology, Johns Hopkins Bloomberg School of Public Health, Baltimore, MD 21205, United States; Department of Health Policy and Management, Johns Hopkins Bloomberg School of Public Health, Baltimore, MD 21205, United States; Department of Health Law, Policy and Management, Boston University School of Public Health, Boston, MA 02118, United States; Department of Health Policy and Management, Johns Hopkins Bloomberg School of Public Health, Baltimore, MD 21205, United States; Department of Health Law, Policy and Management, Boston University School of Public Health, Boston, MA 02118, United States; Department of Health Law, Policy and Management, Boston University School of Public Health, Boston, MA 02118, United States

**Keywords:** wealth inequity, food insecurity, housing insufficiency

## Abstract

The COVID-19 pandemic was characterized by large racial and ethnic inequities in acute material hardships. Pre-pandemic economic conditions, including household wealth, may have contributed to these disparities. We used longitudinal data from the Understanding America Study surveys to (1) describe racial and ethnic differences in pre-pandemic household wealth; and to (2) evaluate the association between pre-pandemic household wealth and acute material hardships during the pandemic. We found large racial and ethnic inequities in pre-pandemic wealth, with 48.3% of non-Hispanic White households reporting wealth greater than $100,000, compared to 16.4% and 29.8% for non-Hispanic Black and Hispanic/Latino households, respectively. Adjusted Poisson regression models clustered by household revealed that, during the pandemic, households with less than $100,000 in pre-pandemic wealth had 1.7-3.0 times higher prevalence of food insufficiency and 1.4-2.0 times higher prevalence of housing insecurity compared with households with more than $100,000 in pre-pandemic wealth. Wealth inequities, which are racially patterned in the United States, shape vulnerability to material hardships such as food insufficiency and housing insecurity during economic crises.

## Introduction

In 2021, Black and Hispanic/Latino households in the United States had approximately one-tenth and one-fifth the wealth of White households, respectively, with the wealth gap widening in dollar amounts since 2019.^1^ Structural racism, through historical and modern socioeconomic policies including slavery, redlining, incarceration, low minimum wage, employment discrimination, and exclusion from social support programs (eg, unemployment insurance), has shaped these wealth inequities.^2-7^

Wealth buffers economic downturns, as households with more wealth have more resources to mitigate negative consequences of economic shocks such as job loss.^8^ In this context, Black, Hispanic/Latino, and American Indian/Alaska Native (AI/AN) peoples disproportionately faced economic harms of the COVID-19 pandemic.^9^ Job loss and reduced wages due to the pandemic were concentrated among low-income workers who were predominantly Black and Hispanic/Latino.^10,11^ Compared with non-Hispanic White households, non-Hispanic Black and Hispanic/Latino households were 68% and 55% more likely, respectively, to experience missed work due to illness or job loss.^12^

Economic shocks, like those driven by the start of the COVID-19 pandemic, lead to increased material hardships such as food and housing insecurity, which also disproportionately burden Black and Hispanic/Latino households.^13^ Material hardships are associated with worse physical and mental health outcomes, including reduced life expectancy and elevated suicide rates.^14-18^ While prior research links wealth with health outcomes^19^ and highlights racial and ethnic disparities in food and housing insecurity during the pandemic,^12,13^ less is known about how wealth shapes material hardships during crises. This study addresses that gap by (1) describing racial and ethnic differences in pre-pandemic household wealth; and (2) measuring the relationship between pre-pandemic household wealth and pandemic-era food insufficiency and housing insecurity.

## Methods

### Study population

We used data from the Understanding America Study (UAS), a longitudinal survey of US households conducted by the University of Southern California Dornsife Center for Economic and Social Research. We measured pre-pandemic wealth using UAS data collected between 2014 and March 1, 2020 during its first 3 fieldings of the core Health and Retirement Study questionnaire (waves 12-14).^20^ Hardships during COVID-19 were assessed by the UAS Understanding Coronavirus in America survey (UAS COVID-19 survey), which collected data biweekly from April 1, 2020, through February 16, 2021, and then monthly through July 20, 2021.^21^

Our primary study sample, used to evaluate the relationship between pre-pandemic wealth and food insufficiency, included participants with household wealth data prior to March 1, 2020, and responses to at least 1 UAS COVID-19 survey wave. Of 6193 eligible participants, we excluded 82 (1.3%) with missing demographic or food insufficiency data, yielding a primary analytic sample of 6111 individuals.

We also evaluated a subsample of participants with housing insecurity data. Of the primary analytic sample, our secondary analytic sample included the 4569 (74.8%) participants (from 3995 distinct households) who responded to at least 1 of the 3 survey questions about eviction or foreclosure risk since August 5, 2020—the period when the UAS COVID-19 survey included these questions.

We applied longitudinal survey weights to each of our samples such that our data were representative of the US adult population. We also applied a subpopulation estimation procedure to define the samples for each analysis. This option appropriately calculates standard errors when survey data are restricted to specific subsamples of interest.

### Exposures

To describe racial and ethnic differences in pre-pandemic household wealth (objective 1), we considered self-reported race and ethnicity as a measure of membership in a marginalized or privileged racial and ethnic group.^22^ We defined this categorical exposure variable according to whether participants identified as each of the following: White, Black (African-American), Native American (AI/AN), Asian (Asian-American), Native Hawaiian or other Pacific Islander, and Hispanic/Latino.

To assess relationships between pre-pandemic household wealth and food insufficiency and housing insecurity during the COVID-19 pandemic (objective 2), our exposure of interest was household wealth at the time of the most recently completed pre-pandemic survey wave. Household wealth data were obtained from UAS survey wave 12 for 1655 participants (27.1% of the primary analytic sample), wave 13 for 2845 participants (46.6% of the primary analytic sample), and wave 14 for 1611 participants (26.4% of the primary analytic sample). We defined household wealth categorically as less than $0 (ie, debts exceed assets), $0 to $25,000, $25,001 to $100,000, and more than $100,000. Missing wealth data were imputed by UAS using sample probabilities, random number draws for bracket imputations, and a hot deck draw for amount imputations.^20^

### Outcomes

Our outcome for objective 1 was pre-pandemic wealth, operationalized as described above for our objective 2 exposure variable. The primary outcome for objective 2 was food insufficiency. Consistent with US Department of Agriculture (USDA) methods for coding survey responses,^23^ we defined this binary variable according to whether participants responded affirmatively during any survey wave to the question, “In the past seven days, were you worried you would run out of food because of a lack of money or other resources?”

The secondary outcome for objective 2 was housing insecurity. Participants were considered to be housing insecure if they ever reported greater than 10% likelihood of eviction or foreclosure in response to the following survey question: “What is the percent chance that you will be evicted, go into foreclosure, or be forced by a landlord to move from your current residence in the next thirty days?” In a sensitivity analysis, we defined housing insecurity more conservatively according to a 50% cutoff in response to this same question. Across each of these definitions, participants were additionally considered to be housing insecure if, in the past month, those who had a mortgage ever reported missing or delaying mortgage payments with permission from their lender to do so. Similarly, those who rented their primary residence were categorized as housing insecure if they reported ever missing or delaying payment of rent or paying less than the full amount in the past month, if they received permission from their landlord to delay or reduce payment of their rent. Participants who, since August 5, 2020, reported being evicted or foreclosed, receiving an eviction or foreclosure notice, or being told by a landlord to move from their residence were also defined as housing insecure.

### Covariates

We decided a priori to adjust the objective 2 regression models for demographic characteristics that potentially confound the relationships between pre-pandemic household wealth and pandemic hardships, including self-reported race and ethnicity, age group, sex, and state of residence. We assessed state of residence as reported in the first UAS survey wave and did not account for interstate relocations during the study period.

### Analysis

We first generated descriptive statistics of the demographic characteristics of our weighted primary analytic sample. We then described the distribution of household wealth in this sample by race and ethnicity, using a $\chi^{2}$ test of independence to identify racial and ethnic differences in pre-pandemic household wealth (objective 1).

We then estimated crude and adjusted prevalence ratios (aPRs) and 95% confidence limits (CLs) to evaluate the relationships between each level of household wealth and the outcomes of food insufficiency and housing insecurity (objective 2), using Poisson regression clustered by household. To examine the robustness of our findings with respect to the survey wave in which household wealth data were obtained, we additionally conducted subgroup analyses by (pre-pandemic) UAS survey wave. All analyses were run in Stata 18.0 (StataCorp, College Station, TX).

## Results

### Sample characteristics

The weighted primary analytic sample was predominantly female (53.0%), non-Hispanic White (60.7%), and less than 60 years of age (69.5%). Of the 6111 participants, 674 (11.0%) reported household wealth less than $0, 1842 (30.1%) reported household wealth between $0 and $25,000, 1087 (17.8%) reported household wealth between $25,001 and $100,000, and 2509 (41.1%) reported household wealth greater than $100,000. Participants in higher household wealth categories were disproportionately older, non-Hispanic White males who had higher household incomes and were less likely to receive SNAP benefits (Table S1). The age distribution in our sample was differential by race and ethnicity, with younger non-Hispanic Black and Hispanic/Latino participants, on average, relative to non-Hispanic White participants.

### Household wealth by race and ethnicity

Non-Hispanic White participants were more likely to report greater household wealth than non-Hispanic Black or Hispanic/Latino participants. A majority of non-Hispanic White participants (48.3%) reported household wealth greater than $100,000, compared to just 16.4% and 29.8% of non-Hispanic Black and Hispanic/Latino participants, respectively (Figure S1). A $\chi^{2}$ value of 688.22 (*P* < 0.001) provides evidence of differences in wealth in the sample by race and ethnicity. We also found evidence of disparities in household wealth by race and ethnicity within age groups, with these inequities widening as age increased (Figure S2).

### Association between pre-pandemic wealth and pandemic hardships

During the COVID-19 pandemic, non-Hispanic Black, non-Hispanic American Indian or Alaska Native, and Hispanic/Latino participants were most likely to report experiencing material hardships, including food insecurity and housing insufficiency (Figure S3).

After adjusting for age group, sex, race and ethnicity, and state of residence, lower levels of household wealth were positively associated with increased levels of food insufficiency. Compared to those with household wealth greater than $100,000, participants with household wealth between $25,001 and $100,000 were 1.68 times (95% CL: 1.32, 2.12) as likely to report food insufficiency, participants with household wealth between $0 and $25,000 were 2.95 times (95% CL: 2.42, 3.59) as likely, and participants with household wealth less than $0 were 2.76 times (95% CL: 2.20, 3.46) as likely (Table 1).

**Table 1.** Relationship between pre-pandemic household wealth and food insufficiency during the pandemic in the weighted primary analytic sample (*N* = 6111).

Pre-pandemic household wealth category	Ever food insufficient during the pandemic (*n* = 1483)	Never food insufficient during the pandemic (*n* = 4628)	Crude prevalence ratio (PR)	Adjusted PR^a^
	*n* (%)	*n* (%)	PR (95% confidence limits)	
Less than $0	246 (36.5)	427 (63.5)	3.38 (2.72, 4.20)	2.76 (2.20, 3.46)
$0-$25,000	752 (40.8)	1090 (59.2)	3.78 (3.15, 4.54)	2.95 (2.42, 3.59)
$25,001-$100,000	214 (19.7)	873 (80.3)	1.82 (1.44, 2.31)	1.68 (1.32, 2.12)
More than $100,000	271 (10.8)	2238 (89.2)	Ref.	Ref.

^a^Adjusted for age group, sex, race and ethnicity, and state of residence.

Among the secondary analytic sample and after adjusting for the same set of covariates used in the previous model, lower levels of pre-pandemic household wealth were also positively associated with increased levels of housing insecurity during the pandemic. Compared to participants with household wealth greater than $100,000, those with household wealth between $25,001 and $100,000 were 1.35 times (95% CL: 1.13, 1.61) as likely to report housing insecurity, those with household wealth between $0 and $25,000 were 2.03 times (95% CL: 1.76, 2.34) as likely, and those with household wealth less than $0 were 2.01 times (95% CL: 1.71, 2.37) as likely (Table 2). Our sensitivity analysis that defined housing insecurity more conservatively produced comparable results, with a slightly greater magnitude of association between household wealth and housing insecurity (Table S2). Both the food insufficiency (Table S3) and housing insecurity (Table S4) findings were robust to variation in the survey wave from which household wealth data were obtained.

**Table 2.** Relationship between pre-pandemic household wealth and housing insecurity during the pandemic in the weighted secondary analytic sample (*N* = 4569).

Pre-pandemic household wealth category	Ever housing insecure during the pandemic (*n* = 1791)	Never housing insecure during the pandemic (*n* = 2778)	Crude prevalence ratio (PR)	Adjusted PR^a^
	*n* (%)	*n* (%)	PR (95% confidence limits)	
Less than $0	308 (54.5)	257 (45.5)	2.38 (2.03, 2.79)	2.01 (1.71, 2.37)
$0-$25,000	796 (56.7)	607 (43.3)	2.48 (2.17, 2.83)	2.03 (1.76, 2.34)
$25,001-$100,000	281 (34.0)	547 (66.0)	1.48 (1.25, 1.76)	1.35 (1.13, 1.61)
More than $100,000	406 (22.9)	1367 (77.1)	Ref.	Ref.

^a^Adjusted for age group, sex, race and ethnicity, and state of residence.

## Discussion

We highlighted racial and ethnic disparities in pre-pandemic wealth, with 48.3% of non-Hispanic White participants reporting household wealth exceeding $100,000, compared to 16.4% of non-Hispanic Black and 29.8% of Hispanic/Latino participants. These disparities likely contributed to the disproportionate burden of material hardships among families of color, as pre-pandemic household wealth was inversely associated with pandemic-era food insufficiency and housing insecurity. Households with less than $100,000 in pre-pandemic wealth displayed 1.7-3.0 times higher prevalence of food insufficiency and 1.4-2.0 times higher prevalence of housing insecurity during the COVID-19 pandemic, compared to households with more than $100,000 in wealth. These findings underscore how racial and ethnic wealth inequities leave Black and Hispanic/Latino households especially vulnerable to losing access to food and housing during crises.

While this study is among the first to examine the relationship between pre-pandemic wealth and pandemic-era material hardships using longitudinal data, our work is consistent with literature on social and political determinants of health that highlights how structural factors shape health outcomes.^24-26^ As such, eliminating wealth-associated health inequities, such as food insufficiency and housing insecurity, will require structural solutions. Future research may address how historical policies, such as slavery,^7^ racial discrimination in housing,^27^ and exclusion of Black people from Social Security programs,^28^ have shaped socioeconomic position over time, and how these practices contributed to exclusion from modern-day social safety-net programs.^29^

Our study has several limitations. First, we use survey data, which is self-reported and inherently subject to limitations such as recall bias. Survey questions that ask participants to report the percent chance that they will be evicted, go into foreclosure, or be forced by a landlord to move from their current residence in the next thirty days are especially subjective and potentially less accurate. Second, the UAS surveys are limited as it is possible for multiple members of a household to respond to the survey and to provide conflicting information about their household's assets with no process for information reconciliation. Third, missing wealth data were imputed by UAS using sample probabilities, random number draws for bracket imputations, and a hot deck draw for amount imputations, although multiple imputation would likely produce more accurate values. Additionally, UAS wealth data were reported across 3 survey waves from 2014 to 2020; although there were no differences in the associations with food insufficiency or housing insecurity over time, the value of our wealth categories and the purchasing power of assets may have changed due to inflation. Last, our analyses are correlational and thus our findings may be subject to unmeasured confounding of the relationship between pre-pandemic household wealth and pandemic-era material hardships.

## Conclusion

We found lower pre-pandemic household wealth was positively associated with pandemic-era food insufficiency and housing insecurity. Moreover, we described substantial racial and ethnic disparities in pre-pandemic wealth. Our analysis highlights how wealth inequities, shaped by deeply rooted and persistent structural racism, may increase vulnerability to material hardships during times of crisis.

## Supplementary Material

qxaf078_Supplementary_Data
