# Supplementary material for: Association between pre-pandemic wealth and material hardships during the COVID-19 pandemic: how racial and ethnic wealth inequities shape household vulnerability to national crises
Source: Health Aff Sch. 2025 Apr 9;3(5):qxaf078. doi: 10.1093/haschl/qxaf078 (PMC12048749; doi:10.1093/haschl/qxaf078)
Supplement: qxaf078_Supplementary_Data [file qxaf078_supplementary_data.zip › UAS Wealth Supplement_HAS.docx]

**Association between pre-pandemic wealth and material hardships during the COVID-19 pandemic**

**Supplementary Table 1.** Demographic characteristics of weighted primary analytic sample by pre-pandemic household wealth category

**Supplementary Figure 1.** Household pre-pandemic wealth distribution by race and ethnicity in primary analytic sample

**Supplementary Figure 2.** Household pre-pandemic wealth distribution by age group and race and ethnicity in primary analytic sample

**Supplementary Figure 3.** Experiences of material hardships during the pandemic by race and ethnicity in weighted primary analytic (food insufficiency) and weighted secondary analytic (housing insecurity) samples

**Supplementary Table 2.** Relationship between pre-pandemic household wealth and housing insecurity during the pandemic, defined more conservatively, in the weighted secondary analytic sample

**Supplementary Table 3.** Relationship between pre-pandemic household wealth and food insufficiency during the pandemic by pre-pandemic survey wave

**Supplementary Table 4.** Relationship between pre-pandemic household wealth and housing insecurity during the pandemic by pre-pandemic survey wave

**Supplementary Table 1.** Demographic characteristics of weighted primary analytic sample by pre-pandemic household wealth category (*N* = 6111)

| **Characteristic** | **Pre-pandemic household wealth** *n* (%) | | | |
| --- | --- | --- | --- | --- |
|  | Less than $0  (*n* = 673) | $0 - $25,000  (*n* = 1842) | $25,001 - $50,000  (*n* = 1087) | More than $100,000  (*n* = 2509) |
| **Sex**  Male  Female | 251 (37.4)  422 (62.7) | 733 (39.8)  1109 (60.2) | 485 (44.7)  602 (55.4) | 1401 (55.8)  1108 (44.2) |
| **Age group**  18 – 29 years  30 – 39 years  40 – 49 years  50 – 59 years  60+ years | 86 (12.8)  249 (37.0)  142 (21.1)  92 (13.6)  104 (15.5) | 407 (22.1)  511 (27.7)  351 (19.0)  259 (14.1)  314 (17.1) | 71 (6.5)  319 (29.4)  215 (19.8)  178 (16.4)  304 (28.0) | 71 (2.8)  413 (16.5)  425 (17.0)  463 (18.4)  1137 (45.3) |
| **Race/ethnicity**  Non-Hispanic White  Non-Hispanic Black  Non-Hispanic American Indian or Alaska Native  Non-Hispanic Asian or Pacific Islander  Non-Hispanic multiple races  Hispanic/Latino | 343 (51.0)  94 (14.0)  3 (0.4)  17 (2.5)  36 (5.3)  180 (26.7) | 872 (47.4)  388 (21.1)  17 (0.9)  50 (2.7)  52 (2.8)  463 (25.1) | 700 (64.4)  128 (11.8)  5 (0.5)  34 (3.2)  28 (2.6)  192 (17.6) | 1792 (71.4)  120 (4.8)  4 (0.2)  150 (6.0)  89 (3.6)  354 (14.1) |
| **Household income**  $0 - $25,000  $25,001 - $50,000  $50,001 - $75,000  $75,001 - $100,000  More than $100,000 | 171 (25.5)  201 (29.8)  133 (19.8)  78 (11.6)  90 (13.4) | 831 (45.1)  490 (26.6)  293 (15.9)  108 (5.9)  120 (6.5) | 196 (18.1)  283 (26.0)  257 (23.7)  153 (14.1)  198 (18.2) | 184 (7.4)  465 (18.5)  506 (20.2)  419 (16.7)  935 (37.3) |
| **Primary residence**  Mortgage  Rent  Neither mortgage nor rent | 236 (35.1)  346 (51.4)  91 (13.5) | 411 (22.3)  1100 (59.7)  331 (18.0) | 622 (57.2)  252 (23.2)  213 (19.6) | 1461 (58.2)  250 (10.0)  798 (31.8) |
| **Employed (as of February 2020)** | 471 (70.0) | 1041 (56.5) | 760 (70.0) | 1592 (63.4) |
| **Ever received SNAP benefits** | 224 (33.2) | 765 (41.5) | 245 (22.5) | 214 (8.5) |
| **Ever received an Economic Impact Payment** | 585 (86.9) | 1439 (78.1) | 971 (89.3) | 2126 (84.7) |

Note: The table presents the weighted percentage of participants in the primary analytic sample who reported each characteristic, stratified by pre-pandemic household wealth category

**Supplementary Figure 1.** Household pre-pandemic wealth distribution by race and ethnicity in primary analytic sample

Note: The figure presents the weighted percentage of non-Hispanic White, non-Hispanic Black, and Hispanic/Latino participants who completed the UAS core questionnaire prior to March 1, 2020, and reported total household wealth within each wealth category at the time the survey was taken.

**Supplementary Figure 2.** Household pre-pandemic wealth distribution by age group and race and ethnicity in primary analytic sample

Note: The figure presents the weighted percentage by age group of non-Hispanic White, non-Hispanic Black, and Hispanic/Latino participants who completed the UAS core questionnaire prior to March 1, 2020, and reported total household wealth within each wealth category at the time the survey was taken.

**Supplementary Figure 3.** Experiences of material hardships during the pandemic by race and ethnicity in weighted primary analytic (food insufficiency) and weighted secondary analytic (housing insecurity) samples

Note: The figure presents the weighted percentage of participants who ever reported food insufficiency or housing insecurity during the COVID-19 pandemic by race and ethnicity.

**Supplementary Table 2.** Relationship between pre-pandemic household wealth and housing insecurity during the pandemic, defined more conservatively, in the weighted secondary analytic sample (N = 4569)

| **Pre-pandemic household wealth category** | **Ever housing insecure during the pandemic**  (n = 1189) | **Never housing insecure during the pandemic**  (n = 3380) | **Crude prevalence ratio (PR)** | **Adjusted PR ^a^** |
| --- | --- | --- | --- | --- |
|  | *n (%)* | *n (%)* | *PR (95% confidence limits)* | |
| Less than $0 | 222 (39.2) | 343 (60.8) | 2.72 (2.18, 3.39) | 2.19 (1.75, 2.74) |
| $0 - $25,000 | 522 (37.2) | 881 (62.8) | 2.58 (2.13, 3.13) | 2.00 (1.63, 2.46) |
| $25,001 - $100,000 | 189 (22.8) | 639 (77.2) | 1.58 (1.25, 2.01) | 1.41 (1.11, 1.80) |
| More than $100,000 | 256 (14.4) | 1517 (85.6) | Ref. | Ref. |

^a^ Adjusted for age group, sex, race/ethnicity, and state of residence

**Supplementary Table 3.** Relationship between pre-pandemic household wealth and food insufficiency during the pandemic by pre-pandemic survey wave ^a^ (N = 6111)

| **Pre-pandemic household wealth category** | **Survey wave 12** (n = 1655) | **Survey wave 13**  (n = 2845) | **Survey wave 14** (n = 1611) |
| --- | --- | --- | --- |
|  | *Adjusted prevalence ratio ^b^ (95% confidence limits)* | | |
| Less than $0 | 2.28 (1.60, 3.24) | 3.09 (2.15, 4.43) | 2.67 (1.81, 3.94) |
| $0 - $25,000 | 2.32 (1.70, 3.17) | 3.63 (2.69, 4.90) | 2.50 (1.67, 3.74) |
| $25,001 - $100,000 | 1.10 (0.73, 1.68) | 2.33 (1.68, 3.24) | 1.37 (0.83, 2.26) |
| More than $100,000 | Ref. | Ref. | Ref. |

^a^ Respondents were invited to participate in UAS survey wave 12 beginning in 2014, wave 13 beginning in 2016, and wave 14 beginning in 2018

^b^ Adjusted for age group, sex, race and ethnicity, and state of residence

**Supplementary Table 4.** Relationship between pre-pandemic household wealth and housing insecurity during the pandemic by pre-pandemic survey wave ^a^ (N = 4569)

| **Pre-pandemic household wealth category** | **Survey wave 12** (n = 1245) | **Survey wave 13**  (n = 2095) | **Survey wave 14** (n = 1229) |
| --- | --- | --- | --- |
|  | *Adjusted prevalence ratio ^b^ (95% confidence limits)* | | |
| Less than $0 | 1.71 (1.28, 2.27) | 2.10 (1.66, 2.65) | 2.37 (1.75, 3.21) |
| $0 - $25,000 | 1.91 (1.51, 2.41) | 2.07 (1.69, 2.53) | 1.89 (1.40, 2.56) |
| $25,001 - $100,000 | 1.16 (0.84, 1.60) | 1.44 (1.14, 1.83) | 1.18 (0.81, 1.74) |
| More than $100,000 | Ref. | Ref. | Ref. |

^a^ Respondents were invited to participate in UAS survey wave 12 beginning in 2014, wave 13 beginning in 2016, and wave 14 beginning in 2018

^b^ Adjusted for age group, sex, race and ethnicity, and state of residence
